# Survey of patient satisfaction with the Breastfeeding Education and Support Services of The Royal Women's Hospital, Melbourne

**DOI:** 10.1186/1472-6963-8-83

**Published:** 2008-04-14

**Authors:** Li Yen Chin, Lisa H Amir

**Affiliations:** 1 Key Centre for Women's Health in Society, University of Melbourne, Melbourne, Australia; 2 Mother & Child Health Research, La Trobe University, Melbourne, Australia

## Abstract

**Background:**

The Breastfeeding Education and Support Services (BESS) is a unit of The Royal Women's Hospital in Melbourne, Australia, staffed by International Board Certified Lactation Consultants (IBCLCs), providing day/short-stay and an outpatient clinic for mothers and infants with breastfeeding problems. It is important to measure women's experience of visiting the service as part of quality assurance. The aim of this project was to conduct an anonymous postal survey of clients' satisfaction with BESS.

**Methods:**

An anonymous survey was posted on 16 November 2005 and again on 31 January 2006, to all women who had attended BESS in September 2005.

**Results:**

The response rate was 60.5% (78/129). Eighty percent (62/78) of respondents attended day-stay, 33% (26/78) attended short-stay and 15% (12/78) attended the outpatient clinic. The percentage of women who responded "strongly agree" to the statement "Overall, I am satisfied with the services" was 49% (35/72) and 50% (6/12) for those who went to day/short-stay and the outpatient clinic respectively. Overall, 56% of all respondents responded that the quality of BESS was "better than expected". The most common breastfeeding problem reported was difficulty attaching the baby to the breast, followed by nipple damage, low milk supply and painful feeding.

**Conclusion:**

BESS seems to have provided a satisfactory service to most clients. Most respondents were clearly satisfied with the support given by the IBCLCs and have also responded that the staff were professional and knowledgeable in their field of work.

## Background

### Breastfeeding clinics

The general aims of breastfeeding clinics are to advise, support and encourage mothers who have left hospital and who wish to breastfeed [1-3]. Breastfeeding clinics in Australia are mostly based in maternity hospitals, predominantly staffed by International Board Certified Lactation Consultants (IBCLCs) [4]. Clinics generally provide consultations with Lactation Consultants who observe breastfeeding problems through several feeds and write plans to guide the mothers when they return home [4].

The Breastfeeding Education and Support Services (BESS) is a unit of The Royal Women's Hospital in Melbourne, Australia, for women experiencing problems with breastfeeding. The unit has developed Clinical Practice Guidelines for the management of breastfeeding problems [5] and Fact Sheets for consumers [6].

In Victoria, it is routine for all new mothers to attend Maternal and Child Health Centres where they are seen by triple-certificate nurses (qualified in nursing, midwifery and maternal and child health) in the local community without charge on a regular basis. Most of the women attending BESS are referred by their Maternal and Child Health Nurse because they require additional help with breastfeeding. As well as referral by a health provider, women can self-refer. Women may have given birth at the Royal Women's Hospital or elsewhere. At the time of this study (2005/6) there were five permanent Midwife/IBCLCs and one General Practitioner/IBCLC working at BESS.

BESS offers services in a day/short-stay clinic (across the road from the hospital) and also in an outpatient clinic (Tuesday afternoons). BESS day/short-stay offers services for mothers and babies up to three months of age. "Day-stay" clients stay from around 9 a.m. until 3 p.m.; "short-stay" clients usually arrive around 11 a.m. for one feed only. Each day (Monday to Friday) usually six infant-pairs spend the day with an IBCLC (three mother-infant pairs for each IBCLC) and one mother-infant pair attends a short-stay appointment with the "duty IBCLC", whose job also includes telephone counselling. Day-stay clients are provided with lunch. The IBCLC takes a history, observes several feeds, helps with positioning and attachment if needed, and provides a written plan for the mother. Women are given handouts or shown videos as appropriate for their needs. One of the hospital obstetric doctors in training attends BESS each day to see women who need to see a medical practitioner and/or need a prescription. Outpatient appointments are 30 minutes (with the General Practitioner/IBCLC or the Midwife/IBCLC) and may be for new clients, follow-up appointments, infants over 3 months or antenatal appointments. The hospital provides these services without charging clients.

According to the BESS database, throughout the whole of year 2005, BESS received 1,137 patients and the total number of visits to BESS was 2,029. The proportion of visits to day-stay, short-stay and the outpatient clinic was 67.6% (1371/2029), 7.5% (153/2029) and 16.1% (326/2029) respectively. The main breastfeeding problems reported were difficulties attaching the baby to the breast, difficulty breastfeeding a neonate and low milk supply (October 2004 to March 2005). Other problems include maternal candidiasis, nipple pain, nipple trauma/cracked nipple and slow feeding of newborn.

### Patient satisfaction

Patient satisfaction assessment is important as a mechanism for quality assurance to ensure standards of health care are achieved and maintained [7]. Readers interested in a systematic review of patient satisfaction are recommended to read the 200-plus page review published in Health Technology Assessment in 2002  [7].

One study has suggested that there are six key aspects of general health care that affects satisfaction – these include medical care and information, food and physical facilities, non-tangible environment, quality of food, nursing care, and visiting arrangements [8]. A well known model that measures consumer satisfaction – the SERVQUAL model – has been used to measure consumer satisfaction in many areas of health as well as the service industry [9-11]. The definitions of the SERVQUAL items – tangibles, reliability, responsiveness, assurance and empathy – are listed in Table 1[9].

**Table 1:** Definition of SERVQUAL variables from [9]

**Variable**	**Definition**
Tangibles	Appearance of physical facilities
Reliability	Ability to perform the promised service dependably and accurately
Responsiveness	Willingness to help customers and provide prompt service
Assurance	Knowledge and courtesy of employees and their ability to convey trust and confidence
Empathy	The firm provides care and individualized attention to its customers

We found only two published studies of satisfaction surveys conducted for a breastfeeding clinic [12,13]. The Richmond Breastfeeding Drop-in Centre, in British Columbia, Canada, operated for three hours per week when it was evaluated in 1995 [12]. The 57 respondents rated their satisfaction as 8.7 on a scale of 1 (lowest) to 10 (highest). The most common reasons for attending were "wanting reassurance, crying/fussy baby and poor latch" [12]. Sixty-eight women responded to the satisfaction survey in Saskatoon, Canada, in 1996 [13]. One hundred percent of the respondents (n = 68) replied they were satisfied with the interpersonal aspects of the services, including how well the Lactation Consultants handled the baby, autonomy in decision-making, and the comfort established with the Lactation Consultant [13]. The "quality of information given, support and encouragement and reassurance, and knowledgeable staff"(p.34) were reasons that respondents claimed to have an effect on whether or not they would recommend the service to others or return to the clinic if needed again [13]. Recently in the UK, women attending a drop-in clinic for infant feeding difficulties reported that they found the expert hands-on, one-to-one support was invaluable [14].

The previous client satisfaction survey at BESS was conducted between July to September 2002 [15]. The survey was distributed to an unknown number of women during their visit to BESS (day-stay only in 2002). A total of 78 surveys were completed and an informal report was compiled for the staff [15]. The majority of respondents agreed that the waiting time to attend the service was satisfactory (92%), the location was convenient (92%), the surroundings were comfortable (92%), the advice offered was achievable (90%), meals were satisfactory (86%), and their breastfeeding problem improved (78%). There was no overall measurement of satisfaction; parity and baby's age were the only sociodemographic variables collected.

### Aim

The aim of this project was to conduct an anonymous postal survey to measure patient satisfaction with the Breastfeeding Education and Support Services at The Royal Women's Hospital.

## Methods

The satisfaction survey was generated from the list of variables mentioned earlier; the relevant items were access, facilities, technical performance, interpersonal skills, and communication. *Access *includes issues such as satisfaction with the location of BESS, the service hours and issues with appointment making. *Facilities *include issues with BESS premises, equipment, rooms and food. *Technical performance of staff *includes the competence and the knowledge of staff. *Interpersonal skills *reflect on the emotional side of the consultation; issues such as encouragement, friendliness, courtesy and others are included in this variable. *Communication *includes how well the staff at BESS communicated and listened to their patients. Negative statements were included in the pilot survey to determine their usefulness (eg. "The staff were unprofessional".). There were 11 items for women who attended day or short-stay, and 6 items for women who attended outpatients only (eg. statement about food service was not included as it was not applicable). (An abbreviated version of all statements can be seen in the tables in the Results section).

Women were asked about their breastfeeding situation and whether they would recommend BESS to other mothers with breastfeeding problems. Three questions asked for written comments on the most satisfactory aspect(s) and least satisfactory aspect(s) of their visit(s) and also any suggestions that they had to improve BESS. The last section of the survey collected demographic characteristics of the women (age, marital status, country of birth, travel time to The Royal Women's Hospital, education level and private insurance status).

Discrepancies between patient expectation and perception of service quality received can be assessed from a survey in a variety of ways: a line scale [7], a numeric scale or a word scale. The line scale has a range of 0% to 200% to represent the percentage of the patient's expectation that was fulfilled during the service. Zero percent represents 'none of [the] expectations were met', 100% represents 'all [the] expectations were met' and 200% means that the patient received more than was expected [7]. The numerical scale from 1 to 9 – 1 represents the service quality being a lot worse than the patient expected, 5 represents the service quality being as the patient expected and 9 represents the service quality being a lot better than the patient expected [16]. The word scale gives five responses to the question regarding how the service quality received compared to the patient's expectation: "much better than I expected," "a little better than I expected," "as I expected," "a little worse than I expected" and "a lot worse than I expected [16]." All three methods were piloted with BESS clients to see which one they preferred.

The survey was piloted with 13 women at BESS day/short-stay clinic or outpatient clinic in October 2005. Eight chose the word scale and five chose the number scale to depict their expectations versus their perceived quality of service (no one chose the line-scale). Two women (15%; 2/13) answered on the "agree"/"strongly agree" end of the scale consistently throughout the survey despite the use of negative statements, implying that these women may not have read the statements closely before answering them. Therefore the final survey used the word scale and negative statements were converted to positive ones (e.g. "The staff were professional").

The survey was sent to all women who attended BESS in the month of September 2005 (n = 129), with a reply paid envelope on 16 November 2005. The response rate was 48% (62/129) in mid-January 2006. A second copy (different coloured paper) was mailed to the 129 women, with a letter thanking the women who had returned the survey previously and to request the women who had not returned a survey to do so as soon as convenient for them.

This project was regarded as a quality assurance activity [17] and did not need to be submitted to The Royal Women's Hospital Research and Ethics Committees (Associate Professor James King, Chair, RWH Research Committee, personal communication, 30 May 2007).

### Analysis

Responses to the survey were entered into a database in EpiData Version 3.1. Data were analysed using EpiInfo Version 3.3.2. Descriptive analysis will be presented.

## Results

The final response rate was 61% (78/129). The majority of clients had been to BESS day-stay (80%; 62/78) (Table 2). Of the 12 clients who said they had been to the outpatient clinic; half (n = 6) had been to day/short-stay as well – i.e. only six attended the outpatient clinic only. Thirty-one percent of clients had an appointment within three days (22/72) and 77% (55/71) of the clients within one week (missing = 1). Most clients (61%; 46/72) travelled for less than 30 minutes to attend BESS. Only six women (8%) said they travelled more than 60 minutes (2 responses were missing).

**Table 2:** Number of clients and mean number of visits to BESS day/short-stay and outpatient clinic*

**Service**	**No. of clients (n = 78)**	***%***	**Mean no. of visits**
Day-stay at BESS	62	*80*	2
Short-stay at BESS	26	*33*	1
BESS Outpatient clinic	12	*15*	2

*Clients could attend more than 1 service

The demographic information of the clients is presented in Table 3. The mean age of the clients was 33 years old (range 18–44). Seventy percent (53/72) had a university degree. Ninety-nine percent (75/76) of the clients were married or in a de facto relationship, 78% (58/74) were born in Australia and 59% (45/76) had private insurance.

**Table 3:** Demographic information

**Variables**	**No. of clients (n = 78)**	***%***
**Age**		
< or = 20 years	3	*4*
21 – 30 years	13	*17*
31 – 40 years	52	*75*
>40 years	3	*4*
(Missing = 2)		

**Highest education level**		
Attended secondary school but did not complete Year 12	5	*7*
Completed secondary school to end of Year 12	6	*8*
Completed a diploma/traineeship/apprenticeship/equivalent	12	*16*
Completed a university degree	53	*70*
(Missing = 2)		

**Marital status**		
Married/de facto	75	*99*
Single	1	*1*
(Missing = 2)		

**Country of birth**		
Australia	58	*78*
United Kingdom	8	*11*
Other*	8	*11*
(Missing = 4)		

**Private insurance**		
Yes	45	*59*
No	31	*41*
(Missing = 2)		

*1 each from China, El Salvador, France, Lebanon, Macedonia, New Zealand, Ukraine and Vietnam

The age range of all the babies at first visit was 4 days to 26 weeks with a median of two weeks; 41% (32/78) were less than two weeks old. The median age of babies was two weeks for day/short-stay (range 4 days to 13 weeks) and five weeks (1–26 weeks) for the outpatient clinic. The majority of babies were born at The Royal Women's Hospital (55%, 43/78), followed by the private hospitals, Frances Perry House (14%, 11/78) and St. Vincent's Private Hospital (13%, 10/78).

The majority of the clients reported difficulties with attaching the baby at the breast (83%, 65/78) (clients could report more than one problem); this was also the most common problem seen in BESS day/short-stay services (89%, 64/72). In contrast, the most common problem seen in clients who only went to the outpatient clinic was nipple/breast thrush (67%, 4/6). See Table 4 for list of breastfeeding problems.

**Table 4:** Proportion of clients with each breastfeeding challenge*

**Breastfeeding challenges**	**No. of clients (n = 78)**	***%***
Attachment difficulties	65	*83*
Nipple damage	33	*42*
Low milk supply	28	*36*
Painful feeding	27	*35*
Slow weight gain for baby	20	*26*
Nipple/breast thrush	17	*22*
Frequent or prolonged feeds	15	*19*
Tongue-tie	9	*12*
Mastitis	7	*9*
Premature baby	4	*5*
Other**	9	*12*

*Clients could report more than 1 problem

** "Flat nipples" (n = 2), nipple shield use (n = 2), "baby thrashing around on/off breast" (n = 1), "nipple vasospasm" (n = 1), "painful engorgement" (n = 1), "small baby" (n = 1), oversupply (n = 1)

At their first visits, most of the babies were fully breastfed (53%, 41/78); 20% (16/78) of the babies were breastfed and given expressed breast milk and infant formula; and 18% (14/78) of the babies were breastfed and given expressed breast milk. At the time when the clients completed the survey, 59% (46/78) of the babies were fully breastfed; 13% (10/78) of the babies were fully infant formula fed; and 11% (9/78) were breastfed and given infant formula. Thus, comparing feeding method at the time of the satisfaction survey with feeding at the first visit, the numbers of fully breastfeeding babies increased by five; the number of babies who were breastfed and given expressed breast milk decreased by nine; the number of babies who were breastfed and given formula increased by 16; and the number of babies who were fully formula fed increased by 10.

Clients' satisfaction with BESS day/short-stay services are presented in Table 5. The majority of clients strongly agreed with positive statements about various areas of the service. Furthermore, 49% (35/72) of the clients said they "strongly agree" and 38% (27/72) said they "agree" to being satisfied overall.

**Table 5:** Levels of satisfaction with services at BESS day/short-stay on Cardigan Street (n = 72)

**Items**	**Strongly disagree n *(%)***	**Disagree n *(%)***	**Neither n *(%)***	**Agree n *(%)***	**Strongly agree n *(%)***
Appointment booked within acceptable timeframe (n = 71)	1 *(1)*	3 *(4)*	4 *(6)*	28 *(40)*	35 *(49)*
Location easy to find	0	4 *(6)*	1 *(1)*	39 *(54)*	28 *(39)*
Staff were professional (n = 71)	0	0	1 *(1)*	28 *(39)*	42 *(59)*
Staff were encouraging (n = 71)	0	4 *(6)*	2 *(3)*	27 *(38)*	38 *(54)*
Staff were courteous (n = 71)	0	0	1 *(1)*	25 *(35)*	45 *(63)*
Given opportunity to discuss concerns (n = 71)	1 *(1)*	4 *(6)*	3 *(4)*	28 *(39)*	35 *(49)*
Satisfied with advice (n = 71)	1 *(1)*	8 *(11)*	3 *(4)*	27 *(38)*	32 *(45)*
Written discharge plan easy to follow (n = 65)	1 *(2)*	4 *(6)*	1 *(2)*	27 *(42)*	32 *(49)*
Written discharge plan achievable (n = 65)	2 *(3)*	5 *(8)*	8 *(12)*	21 *(32)*	29 *(45)*
Had sufficient privacy	1 *(1)*	5 *(7)*	5 *(7)*	33 *(46)*	28 *(39)*
Satisfied with food quality (n = 67)	1 *(2)*	4 *(6)*	3 *(5)*	38 *(57)*	21 *(31)*
**Satisfied overall (n = 71)**	**2 *(3)***	**1 *(1)***	**6 *(9)***	**27 *(38)***	**35 *(49)***

Twenty-two percent (16/72) of clients who attended day/short-stay had waited more than seven days for their appointment, but only 31% (5/16) of these respondents said "neither disagree nor agree", "disagree" or "strongly disagree" to the statement "I was able to book an appointment within an acceptable time frame".

In the outpatient waiting room, ten clients (83%; 10/12) had to wait less than 30 minutes for a consultation, two clients (17%; 2/12) had to wait between 30 to 60 minutes and none had to wait for more than an hour. The majority of clients strongly agreed with statements about the service at the outpatient clinic, and 50% (6/12) strongly agreed to being satisfied overall (refer to Table 6).

**Table 6:** Levels of satisfaction with services at BESS outpatient clinic (n = 12)

**Items**	**Strongly disagree n *(%)***	**Disagree n *(%)***	**Neither n *(%)***	**Agree n *(%)***	**Strongly agree n *(%)***
Appointment booked within acceptable timeframe	0	1 *(8)*	2 *(17)*	6 *(50)*	3 *(25)*
Waiting time acceptable	0	0	0	9 *(75)*	3 *(25)*
Staff were professional	1 *(8)*	0	0	2 *(17)*	9 *(75)*
Felt understood by GP/LC	1 *(8)*	1 *(8)*	0	5 *(42)*	5 *(42)*
Helpful advice/information	1 *(8)*	0	1 *(8)*	4 *(33)*	6 *(50)*
Given opportunity to discuss concerns	1 *(8)*	1 *(8)*	0	6 *(50)*	4 *(33)*
**Satisfied overall**	**1 *(8)***	**0**	**0**	**5 *(42)***	**6 *(50)***

**Figure 1:**
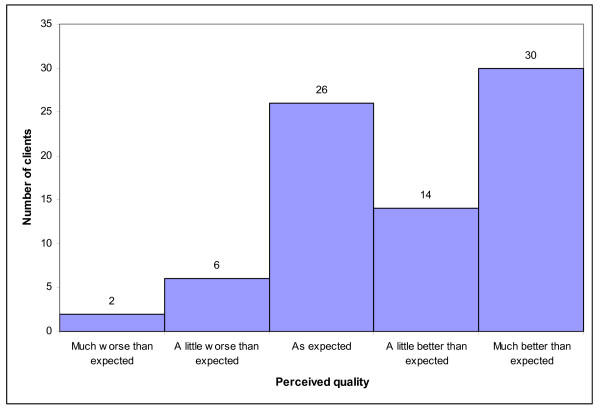
Perceived quality vs. clients' expectation for BESS day/short-stay and outpatient clinic.

The majority of clients who went to BESS found the service quality either as they expected (33%, 26/78) or better than they expected (56%, 44/78). Only 10% (8/78) of the clients said the service quality was worse than they expected (see Figure 1).

Clients were asked how much the visit(s) to BESS helped with their breastfeeding situation; 56% (44/78) of the clients said that the visits to BESS helped "a lot" with breastfeeding the baby, 37% (29/78) either said "partially" or "a little" and 6% (5/78) said "not at all". When asked whether or not clients would recommend BESS to other mothers, 86% (67/78) responded "yes", 12% (9/78) responded "not sure" and 3% (2/78) responded "no".

### Open-text comments

The written comments from the clients were coded and categorized into the themes used in the survey – access, facilities, technical performance of staff, communication with staff and interpersonal relationship.

#### Most satisfactory aspect

A complete list of the categorized comments on the most satisfactory aspect(s) is presented in Table 7. The clients commented most frequently on the positive staff attitude (n = 23).

**Table 7:** Most satisfactory aspects (n = 70)*

**Items**	**No. of clients**
**Access**	
Quick appointment	5
Wonderful resource/Availability of the service	4
Short waiting period	1

**Facilities**	
Comfortable environment	8
Presence of other moms in similar situation	6
Satisfied with food (was provided/quality)	5
Free service	2
Availability of resources	1
Sufficient privacy	1
Follow up contact available	1
Sufficient time in day-stay appointment	1

**Technical performance of staff**	
Staff were professional	18
Knowledgeable staff (satisfied with advice given)	16
Sufficient individual attention	11
Diagnosis – was made/made promptly	4
Written advice (was given/helpful)	2
Solved problem	1
Telephone consultations were helpful	1

**Communication**	
Given options	6
Opportunity to discuss concerns	1

**Interpersonal relationship with staff**	
Good staff attitude	23
Encouragement/Reassurance/Feedback	13
Staff were helpful	1

*Clients could comment on more than one aspect of the visit(s)

"Great support & positive attitude of the midwives, an opportunity to have a rest from daily routine."

"Everything, from the consultation over the phone to in the rooms. The staff were courteous, positive and made me feel at ease, reminding me I wasn't the first or only one!"

In relation to 'access', five clients commented on the availability of quick appointments and also the existence of BESS. For example,

"That I was able to obtain an initial appointment immediately and the follow up appointment was within a more than reasonable time frame. This was very important to me given I was in severe pain and did not know what I could do."

Eight clients liked the comfortable environment that BESS provided. Clients were also pleased with the presence of other mothers in the room (n = 6).

"The clinic is very comfortable."

"The chance to talk with and see other mothers experiencing similar problems."

As part of BESS' service, lunches are provided for clients who are there for day-stay. This was seen as the most satisfactory aspect for some clients (n = 5):

"*...food made you feel nurtured & special."*

The technical performance of staff was also complimented by many clients especially on staff professionalism (n = 18) and knowledge (n = 16):

"*Dedication & patience of consultant top quality."*

"*...detailed knowledge of staff in relation to breastfeeding..."*

"Expert advice that I could privilege over other (conflicting) advice I was receiving in the community."

In the BESS day/short-stay, there is usually one consultant to two to three clients. Even though it was not a one-on-one consultation during the day, some clients (n = 11) still complimented the way the consultants handled the consultations, as seen in the example below:

"The one-on-one service was fantastic, even though there was one consultant for three people, it felt as though it was a one-on-one consultation."

Six clients were most satisfied by the fact that they were given options on how to breastfeed the baby:

"*[I am most satisfied with the] advice on different attachment techniques."*

"Supportive attitude towards formula + expressing, & generally recognizing that WE had to decide what was best for us."

#### Least satisfactory aspect

In Table 8, the categorized comments on the 'least satisfactory aspect(s)' are presented. Some women perceived that the advice was unhelpful (n = 6):

**Table 8:** Least satisfactory aspects (n = 46)*

**Items**	**No. of clients**
**Access**	
Location hard to find/far from residence	2
Long waiting period for day-stay appointment	1
Entrance not welcoming	1

**Facilities**	
Lack of privacy	3
Run down and limitation on facilities/premises	2
Commented on food choices	2
Small premises	1

**Technical performance of staff**	
Non-helpful advice/information	6
Service quality decreases with subsequent visits	2
Staff weren't gentle in handling the baby	2
Lack diagnosis	1
Secondary problem not acknowledged	1
Query about staff qualifications	1
Lack of follow up phone calls	1
Lack of advice	1
Too much advice	1
Not enough help for low milk supply	1

**Communication with staff**	
Lack of staff/individual attention	5
Change of attending staff	3
Mother's feelings were disregarded	2
Insufficient time in day-stay appointment	2
Pushy advice	2
Limited consultation times (for outpatient consultations)	1
Staff weren't attentive to problem	1
Service inequality	1

**Interpersonal relationship**	
Staff were patronizing	1
Lack encouragement	1

*Clients could comment on more than one aspect of the visit(s)

"Did not seem to be helpful with older babies."

"Did not understand the difficulties and difference with twins. All advice felt like it was customised answers for a singleton, no one understood twins."

Other issues that were commented on were the lack of staff and individual attention at the consultation (n = 5) and the lack of privacy (n = 3):

"There were 3 of us in the same room which made it difficult for {the Lactation Consultant} to deal with all of our issues at the same time..."

"*Lack of privacy (sharing room with 2 others) wasn't ideal although not a huge problem."*

The staff at BESS day/short-stay are not on duty every day and thus clients may not see the same consultant on their subsequent visits or on follow-up telephone conversations. This was a least satisfactory aspect for three clients:

"Change of staff. I very much appreciated {one Lactation Consultant's} advice but felt confused by changing to different consultants on subsequent visits."

Two clients felt that their feelings and concerns were disregarded:

"As a previous day stay client I did not feel welcome to ring if I had a concern, on the occasions I did I felt my concerns were dismissed and I was talked at instead of feeling understood."

#### Client suggestions

The suggestions that were made by the clients have been coded and presented in Table 9. The most common suggestion related to the expansion or improvement of BESS premises and facilities (n = 10):

**Table 9:** Suggestions (n = 38)*

**Items**	**No. of clients**
**Access**	
Increase awareness of service availability	4
Improve entrance	1
Prompt service (shorter waiting times)	1

**Facilities**	
Expansion of premises & facilities	10
Improve privacy	2
Home visits	2
Increase staff members	1

**Technical performance of staff**	
Improve advice (balanced advice with bf, ebm and formula)	4
Separate management for older babies	1
Improve advice given through telephone	1
Improve advice given for twins	1
Improve advice on further options	1
Work with other professionals to address secondary complaints	1

**Communications with staff**	
Improve staff communication	1
Promote mother's autonomy over situation	1
Continuity with attending consultant	3

**Interpersonal relationship**	
Give more encouragement	2
Give more empathy to mothers	1

**Other**	
More research into treating low supply	1
"Tell us how to lobby for more resources... "	1

*Clients could have given more than one suggestion

"Expanding the service – shorter waiting periods, more staff, bigger and better facilities however not to the extent that it becomes like a production line and loses its friendliness."

Four clients suggested that BESS increase the awareness of their services:

"*More awareness of this fantastic service for new mums."*

Four clients suggested that staff should give "more balanced" advice on infant feeding:

"Would like to see {lactation consultant} more lenient with what's best for baby + mother – not one vision thinking i.e. MUST breastfeed attitude."

## Discussion

### Limitations

The response rate of 61% was adequate, but we had hoped for a higher response. A higher response may have been achieved if the participants were contacted before the surveys were sent or if telephone contact was used as a follow-up [18]. As we did not have funding for this project, we were limited to a small sample which did not allow any inferential statistical analysis. Although more mothers were fully breastfeeding at the time of the satisfaction survey than they were at their first visit to the clinic, we are unable to state conclusively that attending BESS improved breastfeeding duration as we have no comparison group and we did not follow-up women to see how long they continued to breastfeed.

### Characteristics of respondents and their infants

The mean age of survey respondents was 33 years, which is older than the Victorian average of 30.2 in 2002 [19]. Women attending BESS may be older than average mothers as breastfeeding initiation and duration increases with increasing maternal age [20].

The respondents reported high education levels as well; 68% had completed a university degree. There is a similar finding to the survey from the Saskatoon Breastfeeding Center in Canada, 69% of their respondents reported "having some form of postsecondary education" [13] (p. 34). The women in the UK study were also highly educated; the authors commented that these women tend to have the confidence and motivation to seek out help [14]. In our study, the respondents were mostly born in Australia and married/in a de facto relationship. It is recognised that women with higher education levels, who are married are twice as likely to initiate breastfeeding than single women [20].

In this survey, 41% of infants were less than two weeks old when they first visited BESS, which is less than that reported in the 2002 BESS survey (51%) [15]. Comparisons with the previous BESS satisfaction survey on maternal characteristics and breastfeeding problems are not possible because the previous survey did not collect that information.

### Reported breastfeeding problems

The most common breastfeeding problem reported by the respondents was difficulty in attaching the baby to the breast, followed by nipple damage and low milk supply. This is similar to the primary breastfeeding problems reported in the Saskatoon Breastfeeding Center: the most common was "latch problems" followed by "sore nipples" and "low weight gain/low milk production [13]." Whereas, the most common problems reported the other Canadian breastfeeding centre were "sore, cracked, bleeding nipples" followed by "infant requiring extra fluids" and "very sleepy infant" [1] (p. 395). Twelve percent of survey participants reported infant tongue-tie which is similar to the proportion seen at a breastfeeding clinic in Cincinnati, Ohio (12.8%) [21]. We have previously surveyed clients at BESS whose infants were assessed and/or treated for tongue-tie and found a high level of satisfaction [22].

### Client satisfaction

It is somewhat disappointing that only 56% of women reported that the visit to BESS helped "a lot". Many of the women seen at BESS have complicated breastfeeding problems eg infant tongue-tie as well as maternal low milk supply, or maternal breast infections following complicated birth. In the future, in-depth interviews with women who felt that they were not helped "a lot" by their visit could clarify the reasons for this and offer suggestions for improving the service.

Most clients thought the waiting time for appointments was acceptable. Furthermore, six clients found the quick availability for appointments and the short waiting period the most satisfactory aspects of the service; while only one client found the waiting period for the appointment to be long and the least satisfactory aspect of the service. Similarly, in the 2002 BESS survey, 92% of the respondents reported that the waiting time to attend the service was satisfactory [15].

Most of the clients (78%) agreed with the statement "I had sufficient privacy during my stay" although day-stay clients had to share the room with other mothers. Similarly, 83% of the respondents of the previous BESS survey said they had sufficient privacy in BESS. Many women commented that they enjoyed sharing the room and networking with other mothers [14].

Technical performance and behaviour by health care providers have consistently been related to satisfaction [7]. The respondents were highly satisfied with the technical performance and interpersonal skills of staff members. The satisfaction survey done in the Saskatoon Breastfeeding Center reported that 100% of the responses to satisfaction with the "interpersonal aspects of the service" indicate that the respondents were satisfied with "how the lactation consultant handled the baby," "comfort with the lactation consultant," the autonomy in decision making, the "opportunity to discuss feelings," and the "adequacy of time [13]."

Most of the clients responded that the service quality was as expected or better than they expected. Fulfilled expectation contributes to satisfaction with health care [7], however, the sample size was too small to confirm a relationship between fulfilled expectation and client satisfaction.

### Suggestions

Most of the respondents' suggestions were targeted at the expansion and improvement of BESS facilities. This is an issue that cannot be dealt with easily as it involves high costs and considerable amount of decision-making. Clients also suggested that BESS staff should improve some aspects of the advice they give; this may be achievable with professional development programs for staff. Further patient education materials such as Fact Sheets on breastfeeding twins and older babies could be developed as well.

## Conclusion

Overall, the satisfaction survey showed that most respondents were satisfied with BESS and felt that the service quality was better than they expected. However, due to a mediocre response rate and small sample size, the results may not reflect the true satisfaction level of all BESS clients. In the future, BESS may consider giving clients incentives (e.g. lottery tickets or shopping vouchers) together with the satisfaction survey or use telephone follow-up contact to increase response rates. Future surveys should aim for a larger sample size so that the respondents can better represent all BESS clients and to enable investigation into relationships between breastfeeding outcome and type of breastfeeding problem with satisfaction levels.

In summary, BESS seems to have provided a satisfactory service to most of the clients. Most of the respondents were clearly satisfied with the support given by the IBCLCs and have also responded that the staff were professional and knowledgeable in their field of work. Some clients suggested a few areas where improvements can take place, such as the advice on breastfeeding twins and older babies and promoting the continuity of staff. However, some respondents were unsatisfied with issues such as privacy and also commented on the premises as being old and inadequate. The Royal Women's Hospital is moving in mid-2008, and at the time of writing (September 2007), the location of day/short-stay services of BESS is still undecided.

## Competing interests

LYC declares that she has no competing interests. LHA is employed by the RWH as medical officer in the BESS outpatient clinic (3.5 hours/week).

## Authors' contributions

LYC conducted the project for the Advanced Medical Science year of her medical course at the University of Melbourne. LHA supervised the project and contributed to the writing of the paper.

## Pre-publication history

The pre-publication history for this paper can be accessed here:


